# PPP1R12A is a recycling endosomal phosphatase that facilitates YAP activation

**DOI:** 10.1038/s41598-023-47138-0

**Published:** 2023-11-13

**Authors:** Chiaki Inoue, Kojiro Mukai, Tatsuyuki Matsudaira, Jun Nakayama, Nozomu Kono, Junken Aoki, Hiroyuki Arai, Yasunori Uchida, Tomohiko Taguchi

**Affiliations:** 1 Department of Health Chemistry, Graduate School of Pharmaceutical Sciences, University of Tokyo, Tokyo, Japan; 2 Laboratory of Organelle Pathophysiology, Department of Integrative Life Sciences, Graduate School of Life Sciences, Tohoku University, Sendai, Japan; 3 Laboratory of Integrative Oncology, National Cancer Center Research Institute, Tokyo, Japan; 4 Department of Oncogenesis and Growth Regulation, Research Institute, Osaka International Cancer Institute, Osaka, Japan; 5 Present Address: Division of Molecular Target and Gene Therapy Products, National Institute of Health Sciences, Kawasaki, Japan

**Keywords:** Cell signalling, Organelles, Cell growth, HIPPO signalling

## Abstract

Yes-associated protein (YAP) is a transcriptional coactivator that is essential for the malignancy of various cancers. We have previously shown that YAP activity is positively regulated by phosphatidylserine (PS) in recycling endosomes (REs). However, the mechanism by which YAP is activated by PS in REs remains unknown. In the present study, we examined a group of protein phosphatases (11 phosphatases) that we had identified previously as PS-proximity protein candidates. Knockdown experiments of these phosphatases suggested that PPP1R12A, a regulatory subunit of the myosin phosphatase complex, was essential for YAP-dependent proliferation of triple-negative breast cancer MDA-MB-231 cells. Knockdown of PPP1R12A increased the level of phosphorylated YAP, reduced that of YAP in the nucleus, and suppressed the transcription of CTGF (a YAP-regulated gene), reinforcing the role of PPP1R12A in YAP activation. ATP8A1 is a PS-flippase that concentrates PS in the cytosolic leaflet of the RE membrane and positively regulates YAP signalling. In subcellular fractionation experiments using cell lysates, PPP1R12A in control cells was recovered exclusively in the microsomal fraction. In contrast, a fraction of PPP1R12A in ATP8A1-depleted cells was recovered in the cytosolic fraction. Cohort data available from the Cancer Genome Atlas showed that high expression of *PPP1R12A, PP1B* encoding the catalytic subunit of the myosin phosphatase complex, or *ATP8A1* correlated with poor prognosis in breast cancer patients. These results suggest that the “ATP8A1-PS-YAP phosphatase” axis in REs facilitates YAP activation and thus cell proliferation.

## Introduction

The Hippo pathway is evolutionarily conserved from *Drosophila* to mammals, and plays an essential role in regulating cell proliferation, organ size, and tissue homeostasis^1^. It integrates various extracellular and intracellular cues, including cell–cell contact, mechanical force, and energy status. It consists of the large tumor suppressor LATS1 and LATS2 (hereafter LATS1/2) kinase module and the YAP transcription module^2^. The activity of YAP depends on its phosphorylation status. Unphosphorylated YAP translocates from the cytosol to the nucleus where it binds to TEAD family transcription factors, and induces genes that promote cell proliferation, migration, and survival^3^. In contrast, phosphorylated YAP, generated by active LATS1/2, is sequestered in the cytoplasm and degraded^4,5^. Not surprisingly, dysregulation of the Hippo pathway has been implicated in tumorigenesis in several types of cancers^6^.

Phospholipids are essential components of cellular membranes. PS is the most abundant anionic phospholipid, accounting for up to 10% of the total phospholipids in cells^7^. PS in the plasma membrane (PM) plays key roles in various phenomena such as the coagulation cascade, clearance of apoptotic cells, and recruitment of signalling molecules^8^. PS is also highly enriched in the cytosolic leaflet of the RE membrane through the function of PS-flippase ATP8A1^9^, and regulates endosomal membrane trafficking by recruiting PS-binding proteins, such as evectin-2^10^ and EHD1^9^. Besides its role in membrane trafficking, PS in the RE membrane plays a critical role in YAP signalling^11,12^. Knockdown of ATP8A1 increased the levels of phosphorylated YAP and phosphorylated LATS1 (the active form of LATS1), and abolished the presence of YAP in the nucleus^11^. Furthermore, enhanced PS exposure in REs of alveolar type 2 epithelial cells led to aberrant YAP activation, which may underlie pulmonary fibrosis in Hermansky-Pudlak syndrome (HPS)^13^.

Given the positive contribution of REs to YAP signalling, we reasoned if there were REs-localized protein phosphatases that could dephosphorylate YAP and/or LATS1/2. In the present study, we examined whether the 11 phosphatases, which we had previously identified as PS-proximity protein candidates^11^, contributed to YAP signalling.

## Results

### PPP1R12A is essential for YAP-dependent proliferation of MDA-MB-231 cells

**Figure 1:**
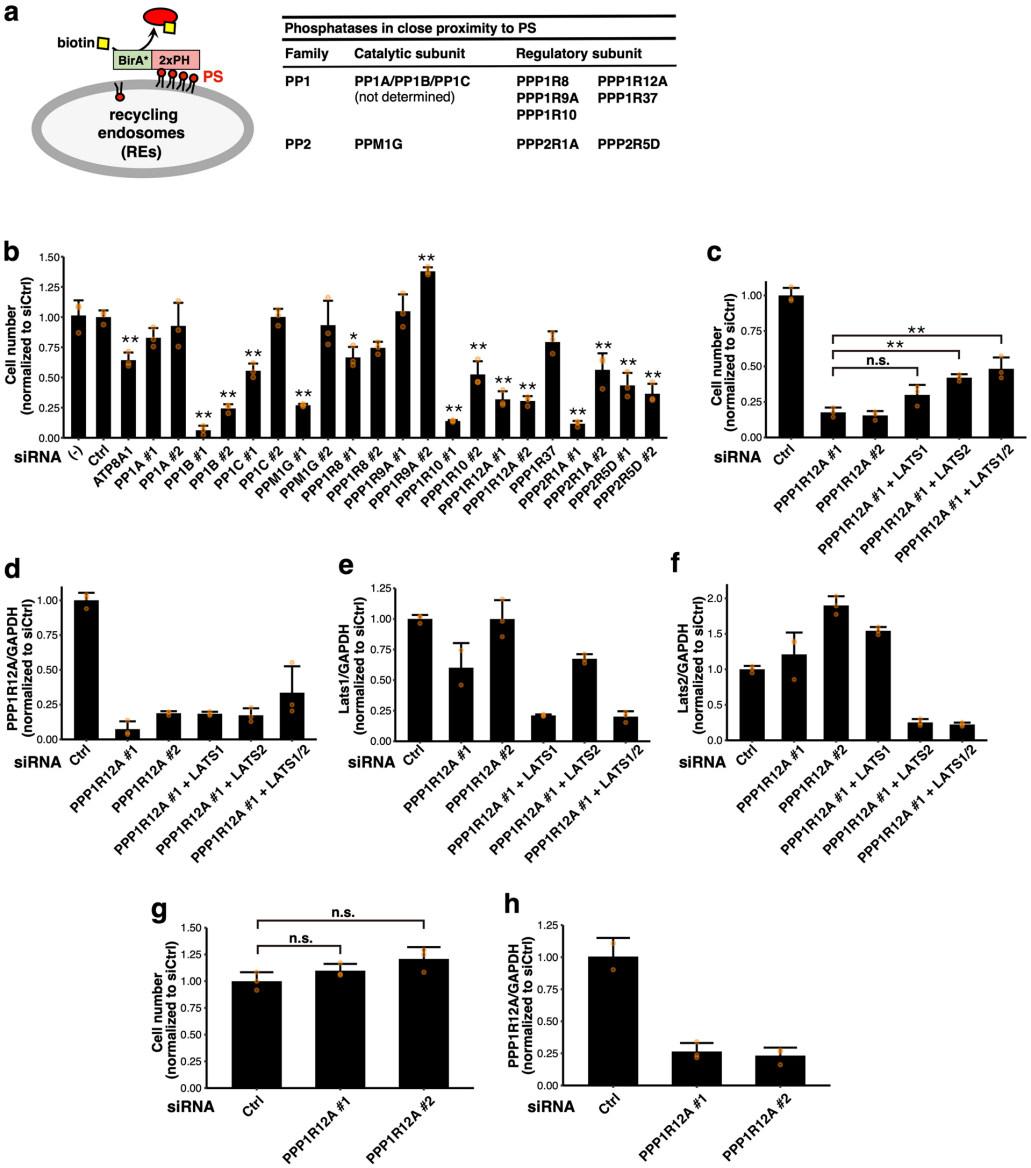
PPP1R12A is essential for the YAP-dependent proliferation of breast cancer cells. (**a**) Schematics of biotinylation of proteins proximal to PS with BirA*-2xPH (the PS biosensor). The list of phosphatases identified by the BioID method using BirA*-2xPH^11^ is shown. (**b**) MDA-MB-231 cells were treated with the indicated siRNA for 48 h and then replated. After 24 h, the cells were transfected with the same siRNA again and further incubated for 96 h. The cell number was counted, normalized to the mean value of control siRNA (siCtrl)-treated cells, and shown in a bar graph. (**c**) MDA-MB-231 cells were treated with the indicated siRNA, and the cell number was counted as in (**b**). (**d–f**) MDA-MD-231 cells were treated with the indicated siRNA as in (**b**), and the mRNA levels of *PPP1R12A* (**d**), *LATS1* (**e**), and *LATS2* (**f**) were determined by qRT-PCR and shown in a bar graph. *GAPDH* was used as an internal control. (**g**) MCF-7 cells were treated with the indicated siRNA as in (**b**), and the cell number was counted, normalized to the mean value of siCtrl-treated cells, and shown in a bar graph. (**h**) MCF-7 cells were treated with the indicated siRNA as in (**b**), and the mRNA level of *PPP1R12A* was determined by qRT-PCR. *GAPDH* was used as an internal control. Data represent the mean ± s.d. from duplicate or triplicate experiments with data points plotted. n.s., not significant; *p < 0.05; **p < 0.01 vs. siCtrl, one-way ANOVA with Tukey–Kramer post hoc test.

To understand the mechanism by which PS in the RE membrane activates YAP, we exploited the list of PS-proximity protein candidates that we had previously identified with the BioID method^11^. The method is based on proximity-dependent biotinylation by a promiscuous bacterial biotin ligase (BirA*) fused to a bait protein^14^. As the bait protein, we had used the tandemly connected pleckstrin homology (2xPH) domain of evectin-2 that specifically binds to PS^9^. Among about 400 biotinylated proteins identified by mass spectrometry, 11 proteins (PP1A, PP1B, PP1C, PPP1R8, PPP1R9A, PPP1R10, PPP1R12A, PPP1R37, PPM1G, PPP2R1A, and PPP2R5D) were the catalytic or regulatory subunits of protein phosphatases (Fig. 1a).

Triple-negative breast cancer MDA-MB-231 cells require YAP signalling for their proliferation^15^. We knocked down these 11 phosphatases individually with siRNA and examined the effect of the knockdown on the proliferation of MDA-MB-231 cells. The results showed that knockdown of PP1B, PPP1R10, PPP1R12A, PPP2R1A, or PPP2R5D significantly suppressed cell proliferation (Fig. 1b). The proliferation defect was confirmed with two independent siRNAs, ruling out the off-target effects. Given that (i) PP1B and PPP1R12A form a phosphatase complex that dephosphorylates various substrates including myosin light chain^16,17^ and (ii) PPP1R12A interacts with YAP in ovarian cancer cell lines^18^, we focused on PPP1R12A in the subsequent experiments.

We sought to confirm if the proliferation defect observed in PPP1R12A-knockdown cells was due to YAP inactivation. To examine this, we knocked down LATS1 and/or LATS2 with PPP1R12A simultaneously. As shown (Fig. 1c), the knockdown of LATS1/2 rescued partially, but significantly, the proliferation defect observed in PPP1R12A-knockdown cells. Quantitative real-time PCR (qRT-PCR) analyses validated the reduced expression of the corresponding genes with siRNA knockdown (Fig. 1d–f). Since the proliferation defect caused by PPP1R12A knockdown was antagonized by LATS1/2 (the YAP inactivating factors) knockdown, PPP1R12A may function as a positive regulator of the YAP signalling.

MCF-7 cells are estrogen-sensitive breast cancer cells. They express YAP at low levels and do not require YAP for proliferation^15,19,20^. As shown (Fig. 1g), knockdown of PPP1R12A in MCF-7 cells did not affect the cell proliferation, albeit the expression of PPP1R12A was efficiently reduced (Fig. 1h). These results supported the specific role of PPP1R12A in YAP-dependent cell proliferation.

### PPP1R12A facilitates dephosphorylation of YAP

**Figure 2:**
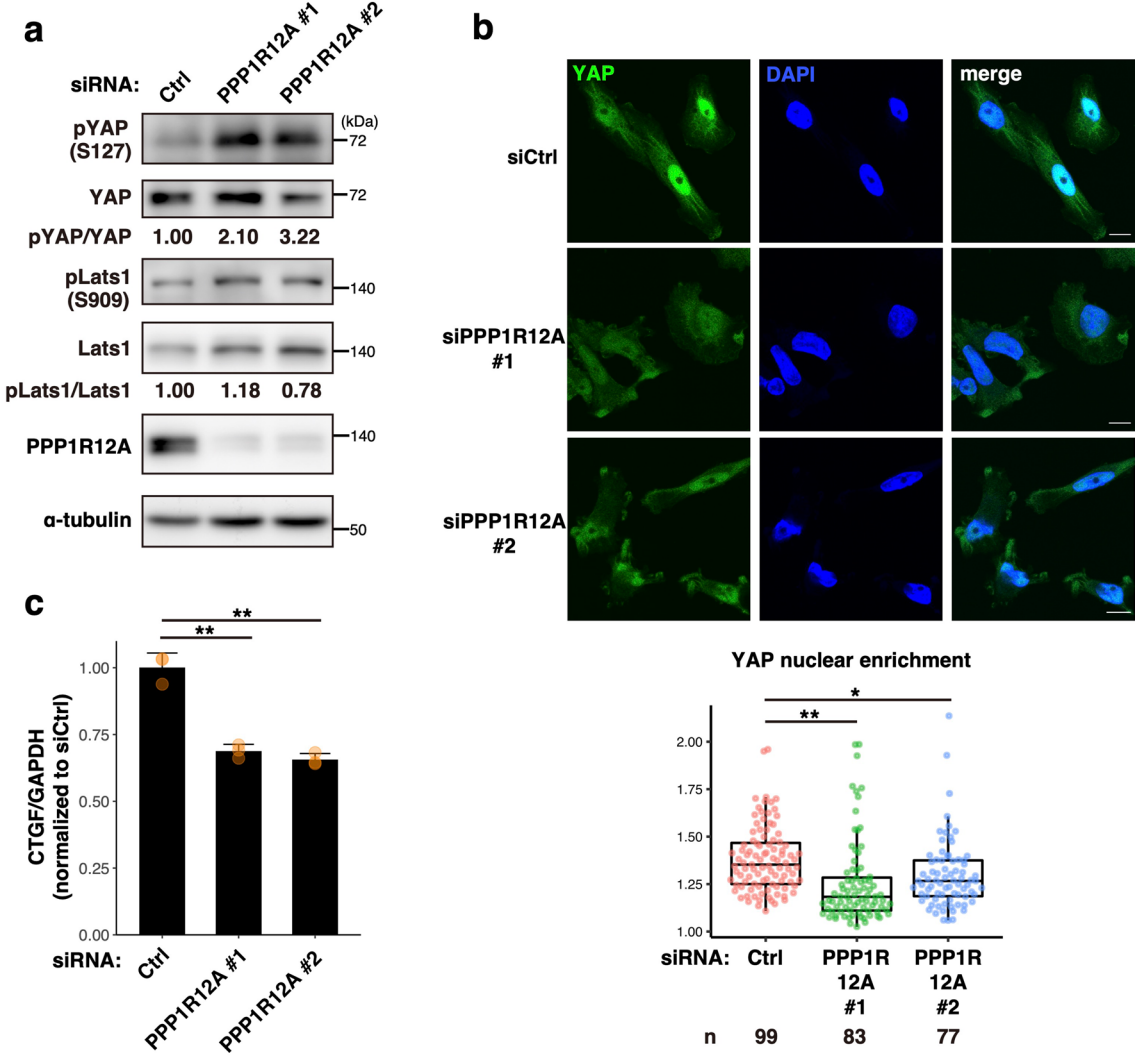
PPP1R12A knockdown increases phosphorylated YAP and reduces the nuclear localization of YAP. (**a**) MDA-MB-231 cells were treated with the indicated siRNA for 48 h and then replated. After 24 h, the cells were transfected with the same siRNA again and further incubated for 48 h. Cell lysates were prepared and analyzed by western blot. (**b**) Cells were treated as in (**a**), fixed, permeabilized, and stained for YAP. Nuclei were stained with DAPI. Scale bars, 10 µm. YAP nuclear enrichment values were determined by calculating the mean nuclear YAP intensity ratio to the mean cellular YAP intensity in individual cells. Data from the indicated number (n) of cells from two independent experiments were shown in box-whisker plots. (**c**) MDA-MB-231 cells were treated with the indicated siRNA for 48 h and then replated. After 24 h, the cells were transfected with the same siRNA again and further incubated for 72 h. The mRNA level of *CTGF* was determined by qRT-PCR. *GAPDH* was used as an internal control. Data are representative of two independent experiments performed in triplicate. The mean values ± s.d. were shown in a bar graph with data points plotted. *p < 0.05; **p < 0.01 vs. siCtrl, one-way ANOVA with Tukey–Kramer post hoc test. The uncropped blots in (**a**) are shown in Supplementary Fig. S1.

We next asked whether PPP1R12A was critical to YAP activation in MDA-MB-231 cells. Knockdown of PPP1R12A increased the inactive form of YAP (the phosphorylated YAP at Ser 127^4^) (Fig. 2a and Supplementary Fig. S1), and significantly reduced the nuclear localization of YAP (Fig. 2b). PPP1R12A knockdown also reduced mRNA expression of CTGF, a YAP-regulated gene involved in cell proliferation (Fig. 2c). These results suggested that PPP1R12A functioned to activate YAP, in line with the notion that PPP1R12A was required for YAP-dependent proliferation of MDA-MB-231 cells (Fig. 1b and c). Although the phosphorylated YAP increased, the active form of LATS1 (the phosphorylated LATS1 at Ser 909^21^) was not affected in PPP1R12A-knockdown cells (Fig. 2a). Thus, PPP1R12A may directly dephosphorylate YAP but not LATS1.

### Endogenous PPP1R12A localizes to REs in MDA-MB-231 cells

Given that PPP1R12A was identified as a PS-proximity protein candidate, PPP1R12A may localize to REs through its binding to PS and mediate dephosphorylation of YAP at REs. Several reports suggested that PPP1R12A resided at various subcellular locations, including the cytosol, nucleus, plasma membrane ruffles, and microfilaments^22–25^, however, its localization in MDA-MB-231 cells has not been examined.

**Figure 3:**
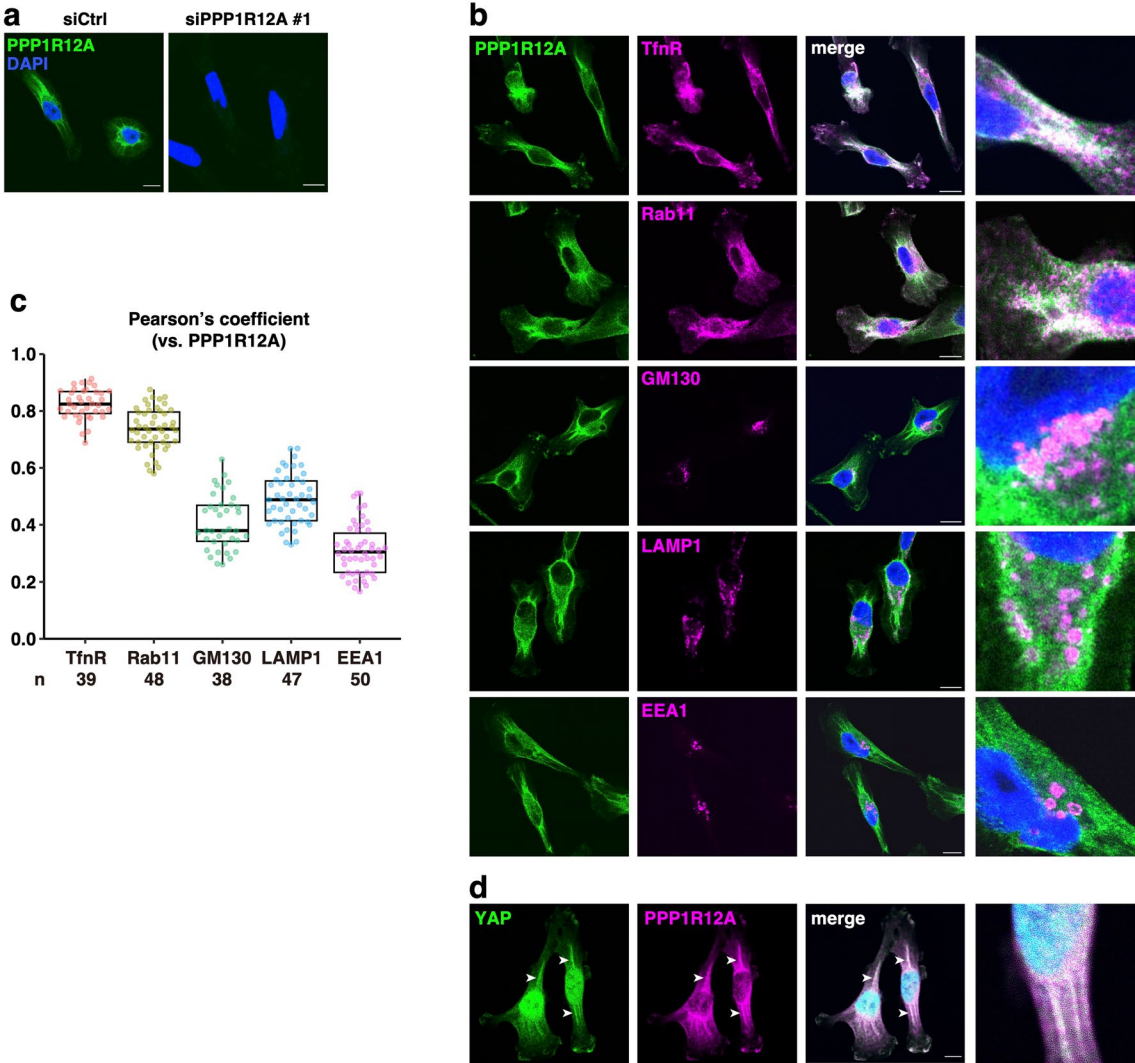
Endogenous PPP1R12A localizes to REs in MDA-MB-231 cells. (**a**) Cells were treated with the indicated siRNA for 48 h and then replated. After 24 h, the cells were transfected with the same siRNA again and further incubated for 48 h. Cells were then fixed, permeabilized, and stained for PPP1R12A. Nuclei were stained with DAPI. (**b**) Cells were fixed, permeabilized, and co-stained for PPP1R12A and the indicated organelle markers. Nuclei were stained with DAPI. (**c**) The Pearson’s coefficient between PPP1R12A and the indicated organelle marker is shown. Data from two independent experiments and the indicated number (n) of cells were shown in box-whisker plots. (**d**) Cells were fixed, permeabilized, and co-stained for PPP1R12A and YAP. Nuclei were stained with DAPI. Arrowheads indicate the co-localization of PPP1R12A and YAP in bundle-like structures. (**b**, **d**) Magnified images of the perinuclear areas were shown in the right panel. Scale bars, 10 µm. Representative micrographs in (**b**) are also shown in Supplementary Fig. S2.

We found that endogenous PPP1R12A localized to perinuclear regions and some bundle-like structures in the cytoplasm (Fig. 3a). These immunoreactive signals were not observed in PPP1R12A-knockdown cells, confirming the specificity of the staining. PPP1R12A co-localized well with the RE proteins, transferrin receptor (TfnR) and Rab11, but not with other organelle proteins [GM130 (Golgi), LAMP1 (lysosomes), and EEA1 (early endosomes)] (Fig. 3b and c, and Supplementary Fig. S2). These results demonstrated that PPP1R12A localized to REs in MDA-MB-231 cells. PPP1R12A also co-localized with YAP in the cytoplasmic bundles (Fig. 3d). Together with the RE localization of PPP1R12A, these data suggested that PPP1R12A interacted with YAP on the RE membrane.

### ATP8A1/PS is involved in the recruitment of PPP1R12A to membranes

**Figure 4:**
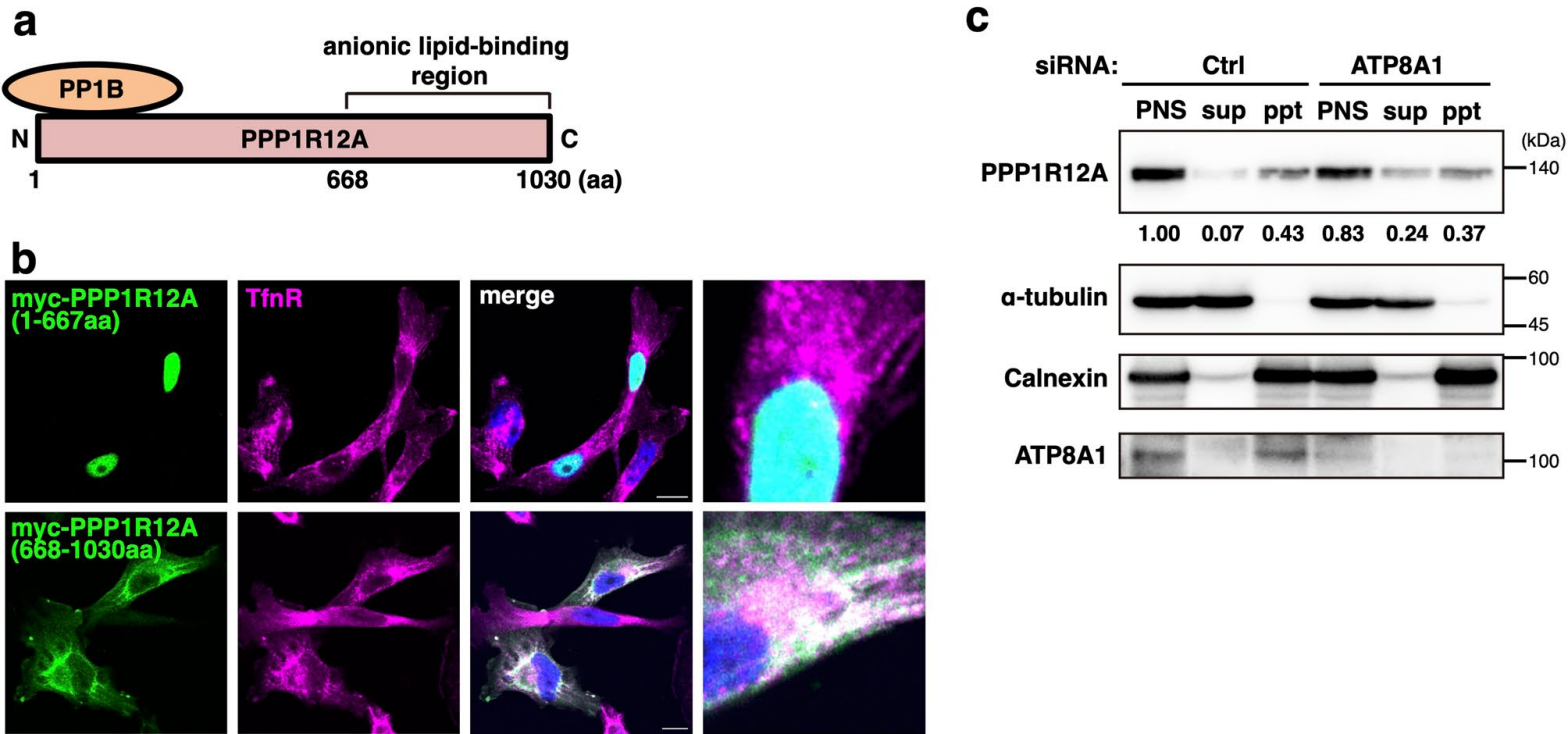
ATP8A1/PS is involved in the recruitment of PPP1R12A to membranes. (**a**) Structure of PPP1R12A. The *N*-terminal region (1–667 aa) binds to PP1B. The *C*-terminal region (668–1030 aa) has an affinity to anionic lipids, such as PS. (**b**) Myc-tagged PPP1R12A truncation mutant (1–667 aa or 668–1030 aa) was transiently expressed in MDA-MB-231 cells. Cells were then fixed, permeabilized, and stained for Myc-tag and TfnR (an RE marker protein). Nuclei were stained with DAPI. Magnified images of the perinuclear areas were shown in the right panel. Scale bars, 10 µm. (**c**) MDA-MB-231 cells were treated with the indicated siRNA for 72 h. The post-nuclear supernatant (PNS) was then spun at 100,000 × g for 1 h at 4 °C. The resultant supernatant (sup), pellet (ppt), and the PNS were subjected to western blot analysis. Data are representative of two independent experiments. The data from the other experiment are shown in Supplementary Fig. S3. The uncropped blots are shown in Supplementary Fig. S1.

Purified recombinant PPP1R12A has been shown to bind anionic phospholipids such as PS, through its *C*-terminal region (Ser 667-Ile 1004 of chicken PPP1R12A, corresponding to Ser 668-Lys 1030 of human PPP1R12A)^26^. PP1B, the catalytic subunit of the phosphatase complex, bound to the *N*-terminal region of PPP1R12A^27,28^ (Fig. 4a). We expressed either the *N*- or *C*-terminal half of human PPP1R12A with a myc-tag in MDA-MB-231 cells and examined their localizations. The *N*-terminal region (1–667) accumulated exclusively in the nucleus, whereas the *C*-terminal region co-localized with TfnR as endogenous PPP1R12A (Fig. 4b). Thus, the *C*-terminal region was essential for PPP1R12A localization to REs.

The ability of the *C*-terminal region to bind anionic phospholipids in vitro may be relevant to PPP1R12A localization to REs. Therefore, we examined if knockdown of ATP8A1, an RE-localized PS flippase, affected the membrane association of PPP1R12A by cell fractionation. In control cells, PPP1R12A was recovered exclusively in the microsomal fraction after ultracentrifugation of the cell lysate, indicating that PPP1R12A was associated with membranes (Fig. 4c and Supplementary Fig. S3). PPP1R12A could not be fully recovered after ultracentrifugation even in the presence of protease inhibitors, indicating that PPP1R12A was unstable in the cell lysate. Nonetheless, in ATP8A1-knockdown cells, the amount of PPP1R12A increased in the cytosolic fraction and decreased in the microsomal fraction. These results suggested that membrane association of PPP1R12A was in part regulated by the levels of PS in the RE membrane.

### Correlation of high *ATP8A1/PPP1R12A/PP1B* expression and poor prognosis in breast cancer patients

**Figure 5:**
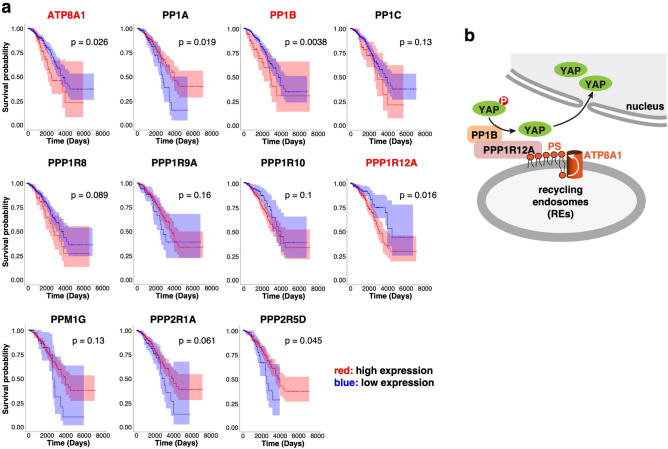
Higher expression of *ATP8A1, PPP1R12A, and PP1B* correlates with shorter survival times in breast cancer patients. (**a**) Kaplan–Meier survival curves based on the expression levels of the indicated gene in cohorts of breast cancer (TCGA, n = 1091) were shown. The colored region along the curve showed the 95% confidence intervals. Number of patients in the each group were represented as *ATP8A1* (High: n = 213, Low: n = 878), *PP1A* (High: n = 884, Low: n = 207), *PP1B* (High: n = 128, Low: n = 963), *PP1C* (High: n = 133, Low: n = 958), *PPP1R8* (High: n = 197, Low: n = 894), *PPP1R9A* (High: n = 948, Low: n = 143), *PPP1R10* (High: n = 800, Low: n = 291), *PPP1R12A* (High: n = 810, Low: n = 281), *PPM1G* (High: n = 982, Low: n = 109), *PPP2R1A* (High: n = 902, Low: n = 189), and *PPP2R5D* (High: n = 944, Low: n = 147). The expression data of *PPP1R37* was not available in TCGA. Significant correlations between high expression levels and shorter survival periods were only observed for *PPP1R12A* and *PP1B* among the genes encoding the PS-proximal phosphatases. (**b**) A model of the regulation of YAP dephosphorylation on the RE membrane.

High *YAP* levels correlate with poor prognosis in triple-negative or estrogen receptor-negative breast cancer patients^29,30^. Therefore, we sought to ask whether *ATP8A1*, *PPP1R12A*, or *PP1B*, which we suggested as YAP-activating factors in the previous and the present study, was related to the prognosis of breast cancer patients. Kaplan–Meier survival curves from TCGA cohort data indicated that high expression of *ATP8A1*, *PPP1R12A*, or *PP1B* correlated with shorter overall survival periods for breast cancer patients (Fig. 5a). In contrast to *PPP1R12A* and *PP1B*, the other phosphatases (*PP1A*, *PP1C*, *PPP1R8*, *PPP1R9A*, *PPP1R10*, *PPM1G*, *PPP2R1A*, and *PPP2R5D*), which were found not to be critical to the proliferation of MDA-MB-231 cells, did not show significant correlations between their high expression levels and shorter survival periods.

## Discussion

In the present study, we showed that PPP1R12A localized to REs (Fig. 3b) and activated YAP in MDA-MB-231 cells (Fig. 2). In contrast, in other cell types, PPP1R12A was shown to inactivate YAP through the activation (or dephosphoryaltion) of Merlin (also known as NF2), the activator of the Hippo pathway^31,32^. The discrepancy may be explained by the fact that MDA-MB-231 cells are homozygous null mutants for Merlin^33^, so that PPP1R12A is unable to activate the Hippo pathway. Of note, Merlin is deleted or mutated in several types of cancers^34^, and thus we suggest that PPP1R12A promotes cell proliferation by acting predominantly on YAP in such Merlin-negative cancer cells.

Incubation of ovarian cancer cell lines with platelets induced the association of PPP1R12A with YAP, coincidentally with YAP dephosphorylation and cellular resistance to anoikis, a type of apoptosis induced by loss of cell adhesion or inappropriate cell adhesion^18^. These observations, together with the present results (Fig. 2a–c), emphasized the role of PPP1R12A in facilitating YAP activation through YAP dephosphorylation. Intriguingly, another Ser/Thr phosphatase PPM1A was recently shown to directly dephosphorylate YAP at Ser 127^35^. Whether there is an interplay between PPP1R12A and PPM1A in the process of YAP activation, remains to be elucidated.

We previously showed that knockdown of ATP8A1 increased the phosphorylation of both YAP and LATS1^11^, contrasting with the present result that knockdown of PPP1R12A increased the phosphorylation of YAP, but not of LATS1 (Fig. 2a). These data suggested that PS in the RE membrane did not only facilitate YAP dephosphorylation by recruiting PPP1R12A to REs, but also regulated LATS1 dephosphorylation by recruiting other unidentified phosphatases. Several PS-proximity phosphatases (PP1A, PP1C, PPM1G, PPP1R8, PPP1R9A, and PPP1R37), the individual knockdown of which did not show any proliferation defect (Fig. 1b), are still such candidates: they may redundantly contribute to LATS1 dephosphorylation.

Rab11, a small GTPase localized to REs, has been shown to transport YAP to epithelial junctions, thereby suppressing YAP translocation into the nucleus and subsequent YAP-dependent transcription^36,37^. A recent study using alveolar type 2 epithelial cells, showed that adaptor protein complex 3 (AP-3)-mediated membrane traffic constitutively transported ATP8A1 out of REs to the lamellar bodies, which resulted in YAP inactivation^13^. Conversely, genetic AP-3 loss in alveolar type 2 epithelial cells and the pearl mouse model caused ATP8A1 retention in REs and concomitant accumulation of PS in the RE membrane and YAP activation, which may underlie progressive lung fibrosis associated with Hermansky-Pudlak syndrome type 2. Therefore, REs can regulate YAP activity, either negatively by membrane traffic out of REs or positively by dephosphorylation of YAP with the RE-localized PS-sensitive phosphatase (Fig. 5b). The balance of these two counteracting factors on REs may determine the phosphorylation/dephosphorylation status of YAP.

Cohort data available from TCGA showed that high expression of *PPP1R12A, PP1B*, or *ATP8A1* correlated with poor prognosis in breast cancer patients (Fig. 5a). Recent studies have shown that high *YAP* expression correlates with shorter survival periods in triple-negative or estrogen receptor-negative breast cancer patients^29,30^. Combined with the present results using MDA-MB-231 cells, the active “ATP8A1-PS-YAP phosphatase (PPP1R12A/PP1B)” axis may account for the poor prognosis of breast cancer patients through YAP activation.

## Methods

### Antibodies

Antibodies used in the present study were as follows: rabbit anti-phospho-YAP (S127) (#13008, dilution 1:500), rabbit anti-phospho-LATS1 (S909) (#9157, dilution 1:500), rabbit anti-YAP (#14074, dilution 1:100 for immunofluorescence), and rabbit anti-PPP1R12A (#8574, dilution 1:1000 for western blot, 1:200 for immunofluorescence) from Cell Signaling Technology; rabbit anti-LATS1 (A300-477A, dilution 1:2000) from Bethyl Laboratories; mouse anti-α-tubulin (10G10, dilution 1:500) from Wako; rabbit anti-Myc (16286-1-AP, dilution 1:800), rabbit anti-calnexin (10427-2-AP, dilution 1:1000), and rabbit anti-ATP8A1 (21565-1-AP, dilution 1:500) from Proteintech; mouse anti-TfnR (H68.4, dilution 1:200), and mouse anti-YAP (sc-101199, dilution 1:200 for immunofluorescence) from Santa Cruz; mouse anti-Rab11 (610656, dilution 1:100), mouse anti-GM130 (610823, dilution 1:500), mouse anti-Lamp1 (555798, dilution 1:500), and mouse anti-EEA1 (610457, dilution 1:200) from BD Biosciences; horseradish peroxidase (HRP)-conjugated goat anti-rabbit or anti-mouse IgG (4050-05, 1031-01, dilution 1:5000), and HRP-conjugated donkey anti-rat IgG (6430-05, dilution 1:5000) from Southern Biotech; Alexa 488- or 594-conjugated secondary antibodies (A21206, A21203, A21202, and A21207, dilution 1:2000) from Thermo Fisher Scientific. Rat anti-YAP (dilution 1:500 for western blot) was a kind gift from Dr. H. Nishina (Tokyo Medical and Dental University, Japan).

### Cell culture

MDA-MB-231 cells and MCF-7 cells were obtained from American Type Culture Collection. MDA-MB-231 cells were cultured at 37 °C in Leibovitz’s L-15 medium (Wako) supplemented with 10% fetal bovine serum (FBS) and penicillin/streptomycin/glutamine (PSG) in a CO_2_-free incubator. MCF-7 cells were cultured at 37 °C in RPMI 1640 medium (Gibco) supplemented with 10% FBS and PSG in a 5% CO_2_ incubator.

### RNA interference

Pre-designed siRNAs used in the study were as follows: control siRNA (Silencer Select negative control No.1, 4390843), PP1A siRNA (#1, s10930; #2, s10932), PP1B siRNA (#1, s10933; #2, s10934), PP1C siRNA (#1, s719; #2, s720), PPM1G siRNA (#1, s10924; #2, s10925), PPP1R8 siRNA (#1, s10954; #2, s10955), PPP1R9A siRNA (#1, s31059; #2, s31060), PPP1R10 siRNA (#1, s328; #2 s329), PPP1R12A siRNA (#1, s9235; #2, s9237), PPP2R1A siRNA (#1, s10963; #2, s10964), PPP2R5D siRNA (#1, s10990; #2, s10991) from Thermo Fisher Scientific; PPP1R37 siRNA (siGENOME M-023860-01) from PerkinElmer. We designed the following siRNA duplexes and purchased them from Nippon Gene: LATS1 siRNA (CACGGCAAGAUAGCAUGGA), LATS2 siRNA (GCCACGACUUAUUCUGGAA), and ATP8A1 siRNA (CUCAAAUGUGGAACGGAUU). Cells were transfected with siRNA (5 or 20 nM) using Lipofectamine RNAiMAX (Thermo Fisher Scientific) according to the manufacture’s instruction.

### qRT-PCR

Total RNA was extracted from cells using Isogen II (Nippon gene), and reverse-transcribed using High-Capacity cDNA Reverse Transcription Kit (Thermo Fisher Scientific). qRT-PCR was performed using SYBR Green PCR Master Mix (Takara) and LightCycler 480 (Roche). The sequences of the primers were as follows. 5′-GCCAAGGTCATCCATGACAACT-3′ (GAPDH, sense primer) and 5′-GAGGGGCCATCCACAGTCTT-3′ (GAPDH, antisense primer); 5′-GCCACCCAGAGACAAGAAAGA-3′ (PPP1R12A, sense primer) and 5′-TAGCCTCTGGTTGTCTGCTTT-3′ (PPP1R12A, antisense primer); 5′-TGGTCATATTAAATTGACTGAC-3′ (LATS1, sense primer) and 5′-CCACATCGACAGCTTGAGGG-3′ (LATS1, antisense primer); 5′-TCATCCACCGAGACATCAAGCC-3′ (LATS2, sense primer) and 5’-TTGTGAGTCCACCTGAACCCAGTG-3′ (LATS2, antisense primer); 5′-GCAGAGCCGCCTGTGCATGG-3′ (CTGF, sense primer) and 5′-GGTATGTCTTCATGCTGG-3′ (CTGF, antisense primer). Target gene expression was normalized to GAPDH content in each sample.

### PCR cloning and plasmid transfection

cDNAs encoding human PPP1R12A (aa 1–667 or 668–1030) were amplified by PCR and then inserted into pMXs-IPuro. The resultant plasmids were transfected by Lipofectamine 2000 (Thermo Fisher Scientific) according to the manufacture’s instruction.

### Cell proliferation assay

Cells were seeded into a six-well plate (1.2 × 10^5^ cells/well). After 24 h, cells were transfected with siRNA and incubated for 48 h. Cells were then replated into 12-well plates in triplicate (5 × 10^4^ cells/well). After 24 h, cells were transfected with the same siRNA again and further incubated for 96 h. The cells were then counted manually or with Countess II FL (Thermo Fisher Scientific).

### Western blot

Proteins were separated in polyacrylamide gel and then transferred to polyvinylidene difluoride membranes (Millipore). These membranes were incubated with primary antibodies, followed by secondary antibodies conjugated to horseradish peroxidase. The proteins were visualized by enhanced chemiluminescence using Fusion SOLO.7S.EDGE (Vilber-Lourmat) or LAS4000 (GE healthcare). Intensity of each protein band was measured with the Gel Analyzer plugin in Fiji (ver. 2.0.0-rc-69/1.52p).

### Immunocytochemistry

Cells were fixed with 4% paraformaldehyde in PBS at room temperature for 15 min and permeabilized with 0.1% Triton X-100 or digitonin (50 µg/ml) in PBS at room temperature for 5 min. Blocking was performed with 3% BSA in PBS at room temperature for 30 min. The cells were incubated with primary antibodies in 3% BSA in PBS or Can Get Signal Immunostain Solution A (TOYOBO) at 4 °C for 16 h, then with secondary antibodies conjugated with Alexa fluorophore in 3% BSA in PBS at room temperature for 1 h. Cells were then mounted with ProLong Glass Antifade Reagent (P36982, Thermo Fisher Scientific).

### Confocal microscopy

Confocal microscopy was performed using LSM880 with Airyscan (Zeiss) with a 20 × Plan-Apochromat dry lens, or a 63 × 1.4 Plan-Apochromat oil immersion lens. Acquired images were Airyscan-processed with Zeiss ZEN 2.3 SP1 FP3 (black, 64-bit) (ver. 14.0.21.201).

### Quantification of imaging data

Imaging data were analyzed with Fiji (ver. 2.0.0-rc-69/1.52p). To quantify YAP nuclear enrichment, the cellular and nuclear masks were created by selecting manually and by thresholding the images of the DAPI channel with Huang’s method, respectively. Mean YAP fluorescence intensities were then measured in these masks. Finally, the YAP nuclear enrichment value was obtained by dividing the mean nuclear YAP intensity by the mean cellular YAP intensity. Co-localization between PPP1R12A and each organelle marker was estimated by calculating Pearson’s correlation coefficient using BIOP JACoP or Coloc 2 plugin in Fiji.

### Subcellular fractionation

Cells were washed with ice-cold PBS twice, and then re-suspended in ice-cold buffer (20 mM Tris–HCl pH 7.4, 250 mM sucrose, 2 mM EDTA, 1 mM DTT) containing protease inhibitor cocktail (25955-11, nacalai tesque). The cells were homogenized with 6 passages through a 27-gauge needle after 6 passages through a 23-gauge needle and centrifuged at 1000 × g for 20 min at 4 °C. The resultant supernatant (post-nuclear supernatant, PNS) was collected and further centrifuged at 100,000 × g for 1 h at 4 °C. The resultant supernatant (cytosolic fraction) and the pellet (microsomal fraction) were subjected to western blot analysis.

### Kaplan–Meier survival analysis

Survival analysis and the log-rank test were performed by R (ver. 3.4.1).

### Statistical analyses

Error bars displayed in bar plots throughout this study represent s.d. from duplicate or triplicate samples. In box-and-whisker plots, the box bounds the interquartile range (IQR) divided by the median, and whiskers extend to a maximum of 1.5 × IQR beyond the box. The corresponding data points are overlayed on the plots. The data were statistically analyzed by performing one-way ANOVA followed by Tukey–Kramer post hoc test with R (ver. 4.1.2) and KNIME (ver. 4.5.1).

### Supplementary Information

Supplementary Information 1.

Supplementary Information 2.

Supplementary Information 3.

## Data Availability

The breast cancer (BRCA) dataset of The Cancer Genome Atlas (TCGA) was downloaded from TCGA Data Portal including clinical information via "RTCGA" R package version 1.16.0. We subjected clinical status and gene expression data to survival analysis. The dataset is available at https://portal.gdc.cancer.gov/. All other data supporting the findings of this study are available from the corresponding author on reasonable request.
