# Supplementary figures and images for: PPP1R12A is a recycling endosomal phosphatase that facilitates YAP activation
Source: Sci Rep. 2023 Nov 13;13:19740. doi: 10.1038/s41598-023-47138-0 (PMC10643656; doi:10.1038/s41598-023-47138-0)

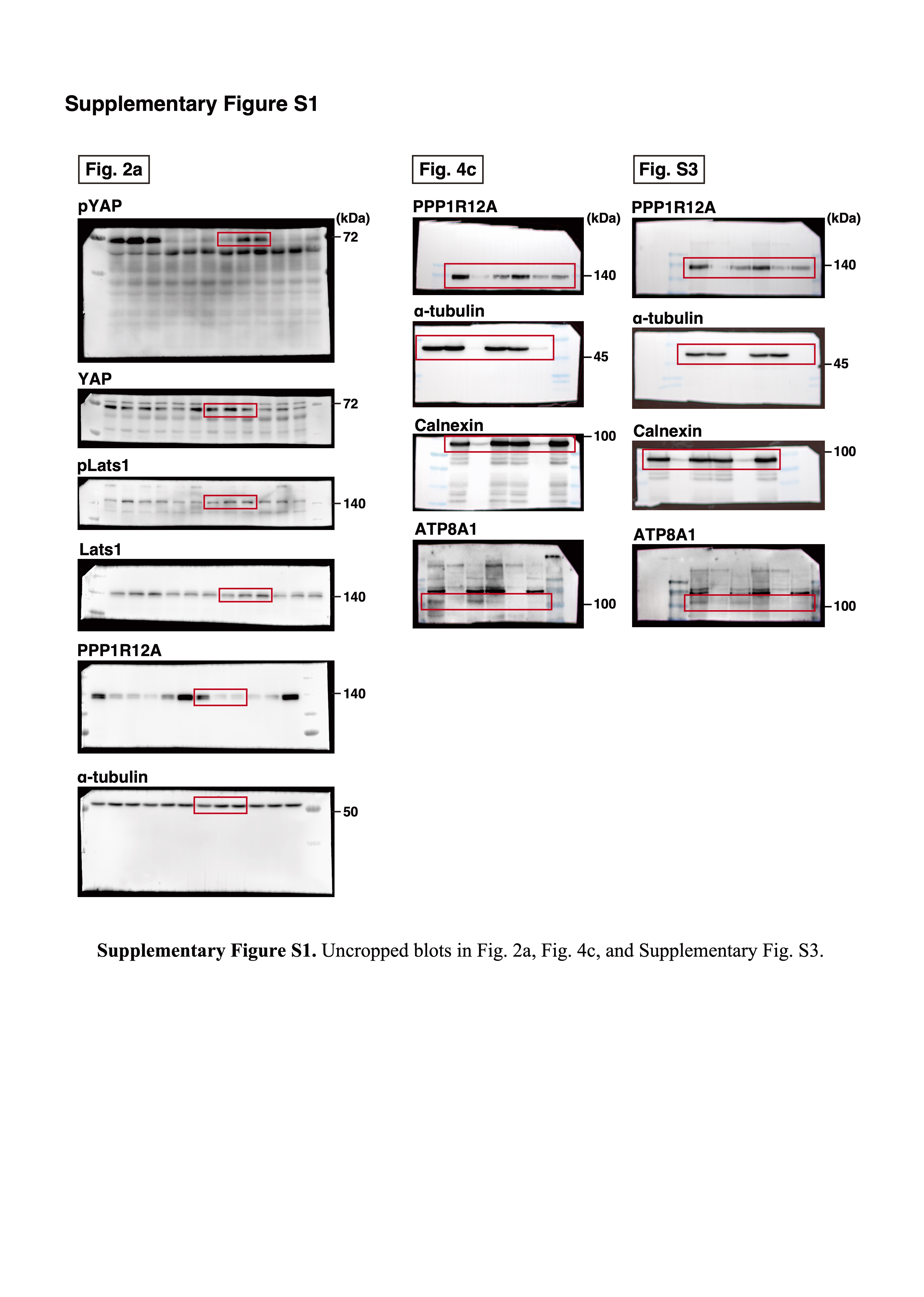

Supplement: Supplementary file 1 — Supplementary Information 1. [file 41598_2023_47138_MOESM1_ESM.jpg]

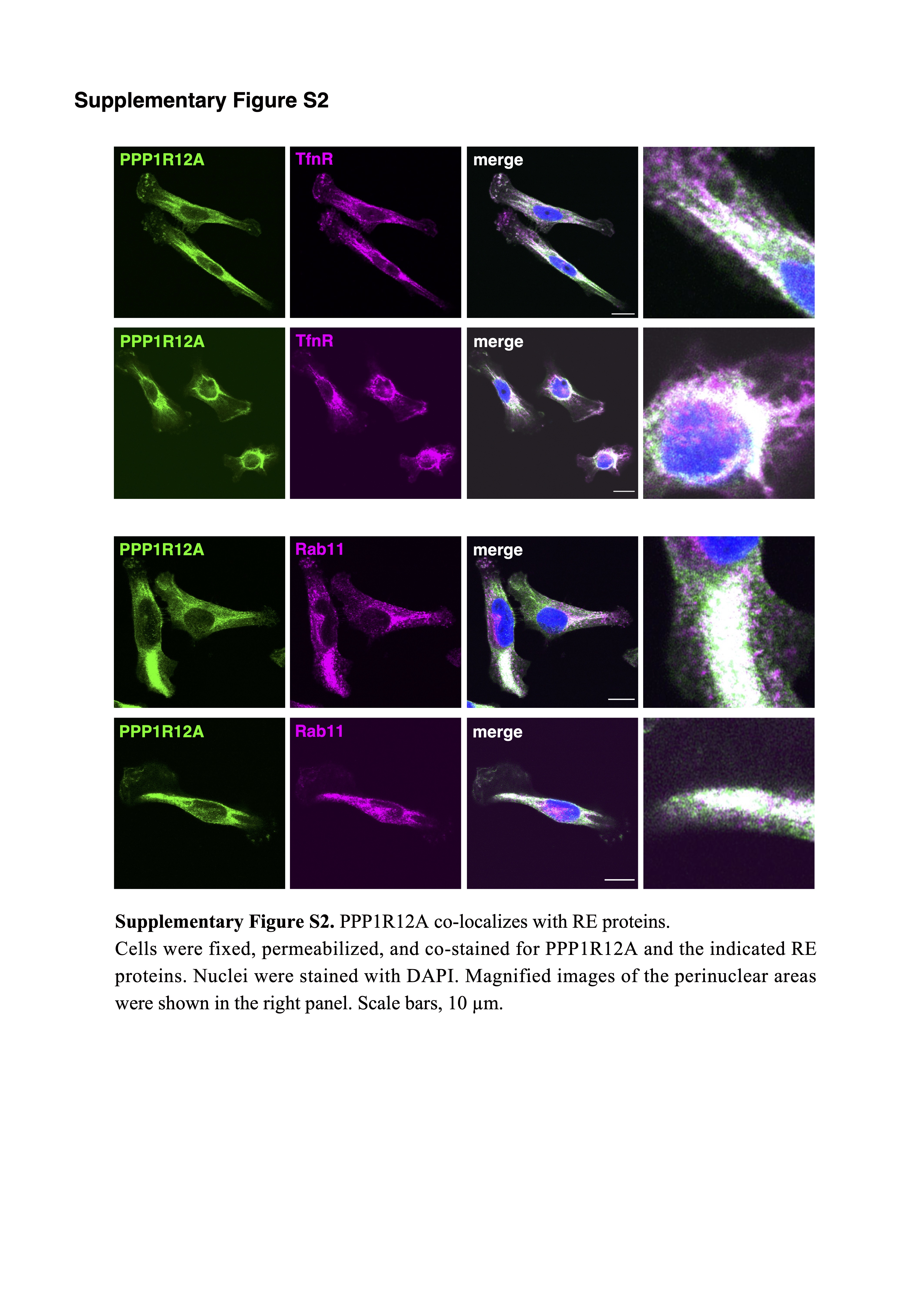

Supplement: Supplementary file 2 — Supplementary Information 2. [file 41598_2023_47138_MOESM2_ESM.jpg]

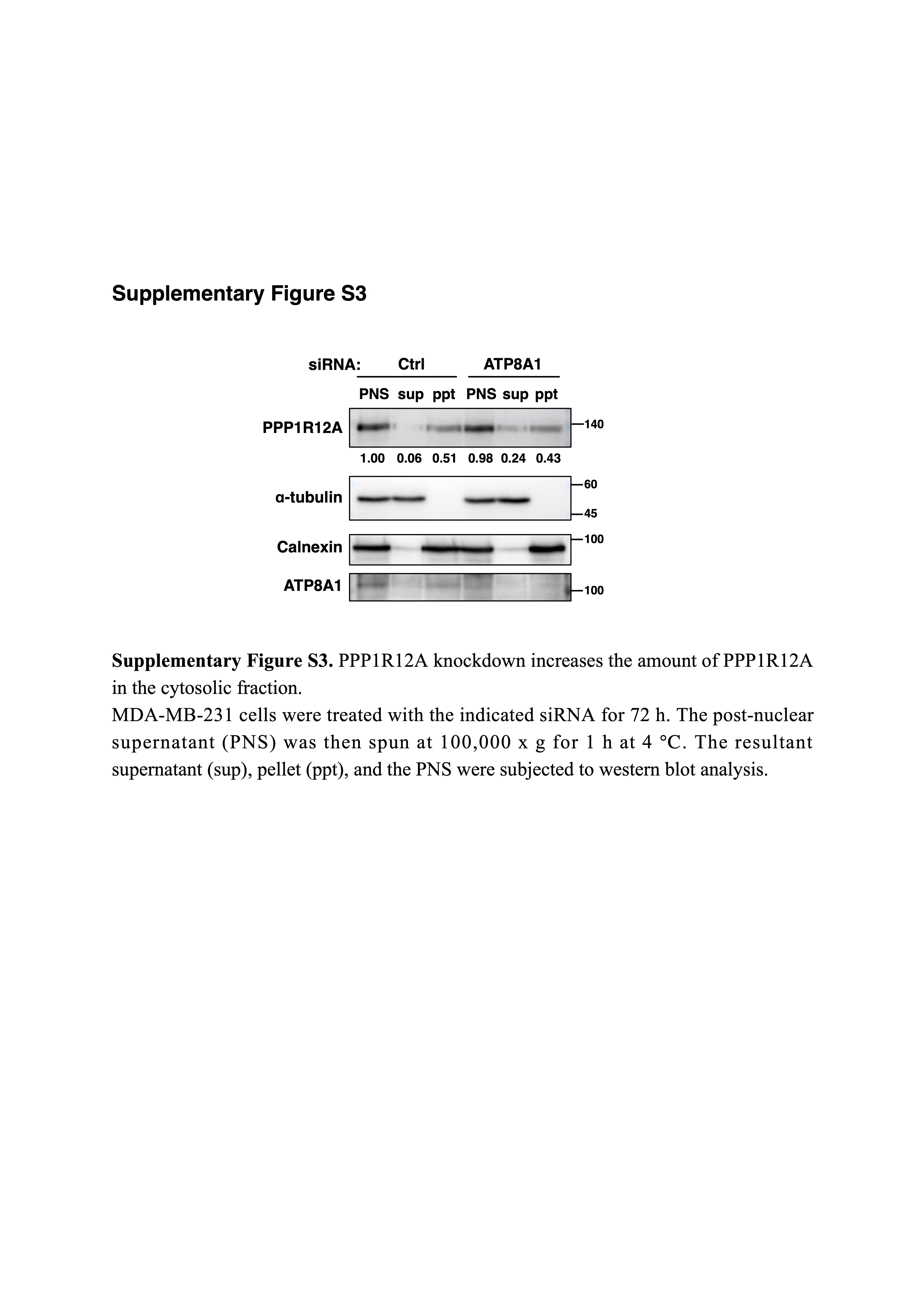

Supplement: Supplementary file 3 — Supplementary Information 3. [file 41598_2023_47138_MOESM3_ESM.jpg]
